# 17β-estradiol and methylprednisolone association as a therapeutic option to modulate lung inflammation in brain-dead female rats

**DOI:** 10.3389/fimmu.2024.1375943

**Published:** 2024-05-03

**Authors:** Marina Vidal-dos-Santos, Lucas F. Anunciação, Roberto Armstrong-Jr, Fernanda Y. Ricardo-da-Silva, Isabella Yumi Taira Ramos, Cristiano J. Correia, Luiz F. P. Moreira, Henri G. D. Leuvenink, Ana C. Breithaupt-Faloppa

**Affiliations:** ^1^ Laboratório de Cirurgia Cardiovascular e Fisiopatologia da Circulação (LIM-11), Instituto do Coração (InCor), Faculdade de Medicina da Universidade de São Paulo, São Paulo, Brazil; ^2^ Department of Surgery, University Medical Centre Groningen, University of Groningen, Groningen, Netherlands

**Keywords:** brain death, females, lung, 17β-estradiol, methylprednisolone

## Abstract

**Introduction:**

Brain death (BD) is known to compromise graft quality by causing hemodynamic, metabolic, and hormonal changes. The abrupt reduction of female sex hormones after BD was associated with increased lung inflammation. The use of both corticoids and estradiol independently has presented positive results in modulating BD-induced inflammatory response. However, studies have shown that for females the presence of both estrogen and corticoids is necessary to ensure adequate immune response. In that sense, this study aims to investigate how the association of methylprednisolone (MP) and estradiol (E2) could modulate the lung inflammation triggered by BD in female rats.

**Methods:**

Female Wistar rats (8 weeks) were divided into four groups: sham (animals submitted to the surgical process, without induction of BD), BD (animals submitted to BD), MP/E2 (animals submitted to BD that received MP and E2 treatment 3h after BD induction) and MP (animals submitted to BD that received MP treatment 3h after BD induction).

**Results:**

Hemodynamics, systemic and local quantification of IL-6, IL-1β, VEGF, and TNF-α, leukocyte infiltration to the lung parenchyma and airways, and adhesion molecule expression were analyzed. After treatment, MP/E2 association was able to reinstate mean arterial pressure to levels close to Sham animals (*p*<0.05). BD increased leukocyte infiltration to the airways and MP/E2 was able to reduce the number of cells (*p*=0.0139). Also, the associated treatment modulated the vasculature by reducing the expression of VEGF (*p*=0.0616) and maintaining eNOS levels (*p*=0.004) in lung tissue.

**Discussion:**

Data presented in this study show that the association between corticoids and estradiol could represent a better treatment strategy for lung inflammation in the female BD donor by presenting a positive effect in the hemodynamic management of the donor, as well as by reducing infiltrated leukocyte to the airways and release of inflammatory markers in the short and long term.

## 1 Introduction

Lung transplantation remains the main option for treating end-stage lung diseases. Even though several surgical teams struggle to reduce the number of patients on the waiting list by seeking strategies to improve lung transplantation, the number of patients with chronic lung diseases continues to rise. In this scenario, the gap between organ necessity and transplants performed will remain a matter of great concern. The majority of organs are procured from brain-dead donors. During the onset of brain death (BD), the loss of the hypothalamic-pituitary axis, and the consequent reduction of several hormones, as well as systemic inflammation and hemodynamic instability have detrimental effects on graft quality (1, 2). Even though there is no consensus regarding the severity of the endocrine compromise in humans; experimental studies in BD models have demonstrated loss of the anterior and posterior pituitary function (3, 4). Clinical studies with hormonal resuscitation, mainly thyroid hormones, vasopressin, and corticoids, have shown positive effects in ameliorating the physiological imbalance after the permanent loss of brain function (5, 6).

In addition, previous evidence indicates that males and females respond differently to the aftermath of BD (7). In experimental models, BD in females is followed by the acute reduction of estradiol (E2) and corticosterone with higher inflammation (8). Treatment of donors with either estradiol or corticoids alone has shown positive effects in experimental and clinical studies of BD (9–15). However, in females, adequate stress response appears to be linked to the presence of both estradiol and corticoids. In rats, elevated levels of corticosterone were observed during periods of higher estrogen concentration and E2 seems to interfere with glucocorticoid release by modulating the autoregulatory capacity of glucocorticoid receptors (GR) (16). In addition, some studies indicate that estradiol receptors (ER) and GR interact with each other (17–19), and could have co-dependent anti-inflammatory actions (20–22). Thus, the sudden lack of these hormones could compromise the female response to BD. We, therefore, aim to investigate the therapeutic potential of E2 and methylprednisolone (MP) association in ameliorating the detrimental effects of BD, focused on the pulmonary inflammatory response in female rats submitted to BD induction.

## 2 Methods

### 2.1 Animals

This study used 52 female Wistar rats (8 weeks). The animals were kept at 23 ± 2°C, 12 h of light and dark periods, without restrictions on water and food intake. Guidelines for animal humane care were in accordance with the ‘‘Principles of Laboratory Animal Care’’ written by the National Society for Medical Research and the ‘‘Guide for the Care and Use of Laboratory Animals’’ published by the Institute of Laboratory Animal Resources from National Institute of Health (NIH Publication No 86-23, revised 1996). Ethical approval for animal experiments was granted by the Faculdade de Medicina da Universidade de São Paulo Ethic Committee for Research Projects (SDC n 1257/2019).

### 2.2 Study groups

**Figure 1:**
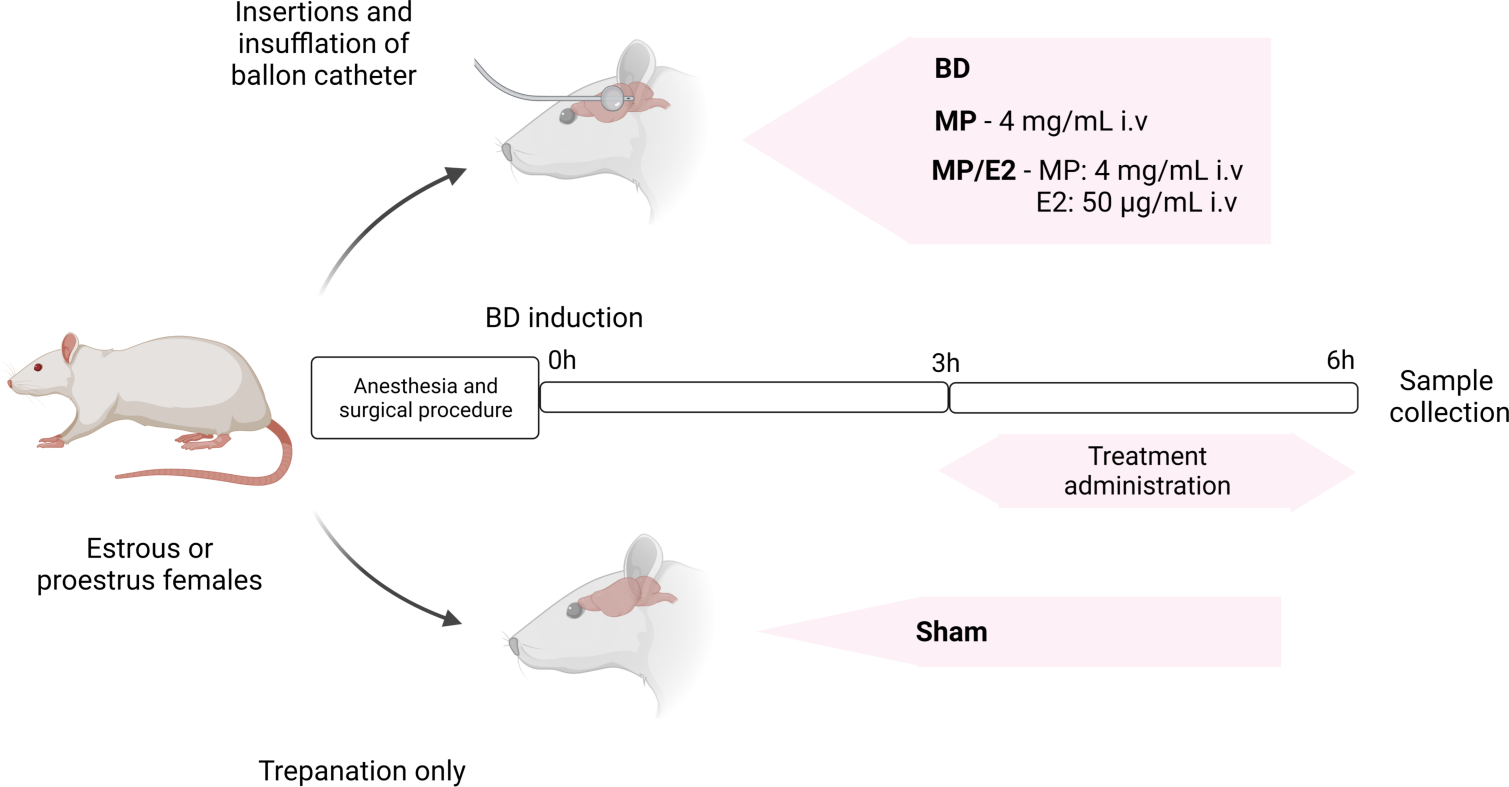
Experimental design of BD induction and treatment administration. BD, brain death. E2, 17β-estradiol. MP, methylprednisolone.

To assure peak estradiol concentration before surgery, animals in the estrus and proestrus phases of the estrous cycle were selected and randomized into four different groups: Sham: rats subjected only to cranial trepanation; BD: rats submitted to brain death; MP: rats submitted to brain death, which received continuous treatment with methylprednisolone after 3h of BD confirmation; MP/E2: rats submitted to brain death, which received a continuous infusion of estradiol and methylprednisolone after 3h of BD confirmation (**Figure 1**).

### 2.3 Anesthesia and induction of brain death

Animals were put under anesthesia with a mixture of isoflurane (5%) and oxygen in a closed chamber, submitted to orotracheal intubation (jelco 16G), and connected to a rodent ventilator (FiO_2_ of 100%, tidal volume of 10 ml/kg, and frequency of 70 cycles/minute). Anesthesia was maintained with 2% isoflurane. Animals were placed on a surgical platform with local heating (37°C) and, after the incision in the anterior cervical region, the right carotid artery was cannulated and connected to a pressure transducer to obtain mean arterial pressure (MAP) values. The right internal jugular vein was also cannulated and connected to an infusion pump for volume replacement and treatment administration. Exposure of the skull cap and total perforation with a spherical drill coupled with a surgical motor in the left parietal region was performed for insertion of a Fogarty^®^ 4F catheter (Baxter Healthcare Co., Marion, NC).

Brain death was induced by rapid infusion of 400 μL of saline solution into the Fogarty^®^ 4F catheter and was confirmed by the hypertensive peak, absence of reflexes, bilateral mydriasis, and apnea. Once brain death was confirmed, anesthesia was discontinued, volume replacement was initiated, and mechanical ventilation was maintained for 6h. Sham animals were kept under anesthesia with isoflurane (2%) until the end of experiments.

### 2.4 Treatment

In the initial 3 h, all animals received a continuous infusion of saline solution (NaCl 0.9%, 2 ml/h). After 3h of BD confirmation, the MP group received continuous infusion (2 ml/h) of methylprednisolone alone (4 mg/mL, i.v) Solu-Medrol®, Pfizer, USA. The MP/E2 group received continuous infusion (2 ml/h) of 17β-estradiol (50 μg/mL, i.v. - Sigma-Aldrich^®^, USA) and methylprednisolone (4 mg/mL, i.v – Solu-Medrol^®^, Pfizer, USA). Sham and BD groups received an equivalent dose of 17β-estradiol dilution vehicle (cyclodextrin) (Sigma-Aldrich^®^, USA) in saline solution (NaCl 0.9%, 2 ml/h).

### 2.5 Determination of hormones serum levels

Blood samples were collected at the end of the sixth hour from the abdominal aorta. Quantification of circulating concentrations of 17β-estradiol and corticosterone was performed using ELISA kits (Cayman Chemical Company, USA) in accordance with the manufacturer’s recommended protocol.

### 2.6 Total and differential cell count on bronchoalveolar lavage

After euthanasia, the bronchoalveolar space was washed with DMEM (5 mL) through the orotracheal cannula. Bronchoalveolar lavage fluid (BAL) was centrifuged (200×g, 15° C. for 10 minutes) and the cell pellet was resuspended in PBS (1 ml). 20 uL of the resulting cell suspensions were used for analyses with an automated hematology analyzer (Mindray BC 2800 Vet, Shenzhen, China).

### 2.7 Isolated tissue culture (explant)

After the desired time elapsed after BD (6 h), lung tissue fragments were incubated in 4-well plates and maintained in a humid atmosphere for 24h with 95% O_2_ and 5% CO_2_ at 37°C in DMEM culture medium (Dulbecco’s Modified Eagle’s Medium, Vitrocell Embriolife, Brazil). The culture medium was collected and stored at -80°C until analyses and lung fragments were placed to dry in an incubator at 37°C and were later weighed.

### 2.8 Homogenization of lung tissue

Lung fragments were weighed and dissociated in PBS (4 µL/g) with GentleMACS Dissociator (Miltenyi Biotec, Germany). The homogenate samples were stored at -80 ° C until analyses.

### 2.9 Determination of inflammatory mediators’ concentration in serum, lung tissue homogenate, and lung culture samples

To determine the concentration of inflammatory mediators in serum (IL-1β, IL-6, VEGF, and TNF-α), lung homogenates supernatants (IL-6, IL-1β, VEGF and TNF-α), and in lung explant medium (IL-6, IL-1β, VEGF and TNF-α) ELISA commercial kits (Duo Set, R & D System^®^, USA) were used in accordance to the manufacturer’s instructions. Optical density was obtained by spectrophotometry (SpectraMax^®^ PLUS Microplate Reader, Molecular Devices, USA). Concentration values were presented as pg/ml for serum, as pg/mg of total protein level for lung homogenates, and as pg/ml/mg of dry weight for explant.

### 2.10 Real-time PCR for gene expression analysis of IL-1β, IL-6, VEGF, TNF-α, eNOS, iNOS, and ICAM-1

Gene expression was quantified by using real-time PCR in a Step One Plus^®^ device (Applied Biosystem, USA). RNA extraction from tissues (lung) was performed using a commercial mirVana™ miRNA isolation Kit (Ambion^®^-Thermo Fisher Scientific, USA), following the manufacturer’s protocol. The cDNA was transcribed (High capacity reverse transcriptase kit, Applied Biosystem, USA) and the real-time PCR reaction was performed. The primers used were Taqman (Applied Biosystem, USA) for GAPDH, β-actin, iNOS, eNOS, VEGF, and ICAM-1 and SYBR^®^Green (Applied Biosystems) for β-actin, IL-1β, IL-6 and TNF-α (**Table 1**): Cycling conditions were as follow: 2 min at 50°C, 10 min at 95°C followed by 40 cycles of 15 sec 95°C and 1 min at 60°C.

**Table 1:** RT-PCR primers used for analysis.

Real-time PCR Taqman		
GAPDH	Rn01775763_g1	
β-actin	Rn00667869_m1*	
iNOS	Rn00561646_m1*	
eNOS	Rn02132634_s1*	
VEGF	Rn 01511601_m1	
ICAM-1	Rn005642227_m1*	
Real-time PCR SYBR^®^Green		
β-actin	RN b-act fw	5´-GGAAATCGTGCGTGACATTAAA-3´
	RN b-act rv	5´-GCGGCAGGGCCATCTC-3´
IL-1β	RN IL-1B fw	5´-CAGCAATGGTCGGGACATAGTT-3´
	RN IL-1B rv	5´-GCATTAGGAATAGTGCAGCCATCT-3´
TNF-α	TB TNF- α fw	5´-AGGCTGTCGCTACATCACTGAA-3´
	RN TNF- α rv	5´-TGACCCGTAGGGCGATTACA-3´
IL-6	RN IL-6 fw	5´-CAACTTCCAATGCTCTCCTAATG-3´
	RN IL-6 rv	5´-TTCAAGTGCTTTCAAGAGTTGGAT-3´

RT-PCR, real-time polymerase chain reaction; GAPDH, glyceraldehyde-3-phosphate dehydrogenase; iNOS, inducible nitric oxide synthase; eNOS, endothelial nitric oxide synthase; VEGF, vascular endothelial growth factor; IL, interleukin; TNF- α, tumor necrosis factor-alpha; ICAM-1, intercellular adhesion molecule 1.

### 2.11 Nitrates and nitrites (NOx^–^) quantification in serum, tissue homogenate, and explant

Lung tissue homogenate, explant, and serum samples were incubated with nitrate reductase (Sigma-Aldrich^®^, USA) for 2 h at 37°C for the reduction of nitrate (NO_3_^-^) into nitrite (NO_2_^-^). After reduction, nitrite detection was performed by incubating the samples with Griess reagent, producing a colorimetric reaction with a wavelength reading of 595 nm. The concentration values were obtained against a NaNO_2_ standard curve (5-60µM). Values are presented as mM/mL in serum and homogenate samples and as nM/ml/mg of dry weight in the explant.

### 2.12 Immunohistochemistry of MPO, ICAM-1, eNOS, and iNOS

The left pulmonary lobe was insufflated with Tissue-Tek^®^ O.C.T. Compound (^©^ Sakura Finetek, USA) through the left bronchus and snapped frozen in a nitrogen-hexane solution. Cryosections (10 μm) were fixated on a glass slide for 10 min in cold acetone. Endogenous peroxidase blockage (H2O2, 2%) was performed. Albumin-rich solution was used for blocking non-specific sites.

Before staining, cryosections were incubated with primary antibodies at TBS-T/BSA overnight at 4°C. Primary antibodies (Boster, 1:100) were used for Mieloperoxidase (MPO) and Intercellular adhesion molecule 1 (ICAM-1), and primary antibodies (Abcam, 1:100) for eNOS and iNOS immunodetection. Sections were then incubated in HRP-conjugated secondary antibodies and later in a peroxidase substrate. 10 images per section were acquired using a DS-Ri1 digital camera connected to an image acquisition system. Analyses were performed using NIS-Element-BD (Nikon, Japan) software. MPO and iNOS results are presented as stained cells per mm^2^. ICAM-1 and eNOS results are presented as stained area per total area, and Vascular cell adhesion molecule 1 (VCAM-1) is presented as stained area per vessel area.

### 2.13 Analysis of results

The results are expressed as mean ± standard error of the mean (SEM) or as median and interquartile interval. Statistical analyses were conducted using GraphPad Prism Software v.9.1.0. The data were analyzed for distribution with a normality test and submitted to analysis by Kruskal-Wallis followed by the post-hoc test of two-stage linear step-up procedure of Benjamini, Krieger, and Yekutieli, always compared to the BD group. MAP mixed effect analysis was performed followed by post-hoc test of the two-stage linear step-up procedure of Benjamini, Krieger, and Yekutieli.

### 2.14 Euthanasia

After 6 hours, animals submitted to BD were exsanguinated through the abdominal aorta. Sham animals were euthanized by exsanguination under anesthesia. Animals were disposed of according to current standards for incineration.

## 3 Results

### 3.1 Hormonal profile

**Figure 2:**
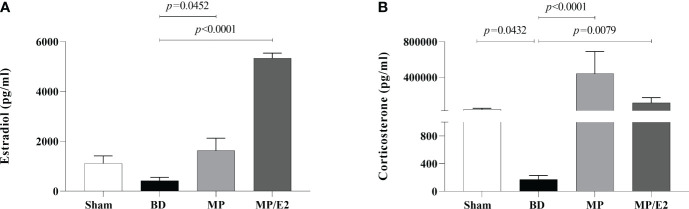
Serum estradiol **(A)** and corticosterone **(B)** concentrations. Sham, false-operated rats; BD, rats submitted to brain death; MP, rats treated with methylprednisolone (MP) after 3h of confirmation of BD and MP/E2, rats treated with 17β-estradiol (E2) and methylprednisolone after 3h of confirmation of BD. Data expressed as mean ± SEM from 8 animals. **(A)** p^(Kruskal Wallis)^=0.0005, **(B)** p^(Kruskal Wallis)^=0.0005.

Data on serum concentration of estradiol and corticosterone showed that both hormones were reduced in the BD group in comparison to Sham animals. Elevated levels of corticosterone were present in both MP/E2 and MP-treated groups, while estradiol increase was only observed in the MP/E2 group (**Figure 2**).

### 3.2 Mean arterial pressure

**Figure 3:**
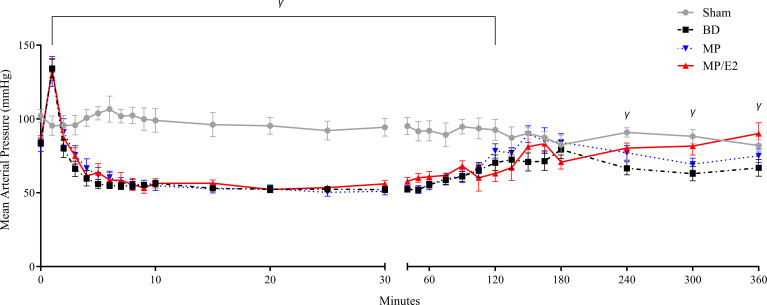
Mean arterial pressure measurements. Sham, false-operated rats; BD, rats submitted to brain death; MP, rats treated with methylprednisolone (MP) after 3h of confirmation of BD and MP/E2, rats treated with 17β-estradiol (E2) and methylprednisolone after 3h of confirmation of BD. Data expressed as mean ± SEM from 8 animals. ^γ^p^(Mixed effect)^<0.05 in relation to the BD group.

Sham animals presented stable MAP during the 6h of experiments. BD resulted in a transient hypertensive crisis accompanied by a period of hypotension and, lastly, normalization of MAP. No significant difference was observed in the group treated with MP. On the other hand, the MP/E2 group, in comparison to the BD group, presented a significant augmentation of MAP after 4h of BD (**Figure 3**).

### 3.3 Serum quantification of inflammatory markers

To evaluate systemic inflammation, several markers were quantified in serum samples. Significant lower levels of IL-6 were observed in both treated groups in comparison to BD. Regarding VEGF, there was a reduction in the BD group compared to Sham, and even lower levels were found with the associated treatment (MP/E2) in comparison to BD. None of the other markers analyzed presented significant differences among the groups (**Table 2**).

**Table 2:** Quantification of inflammatory mediators (pg/mL) in the serum of rats submitted to BD. Sham, false-operated rats; BD, rats submitted to brain death; MP/E2, rats treated with 17β-estradiol (E2) and methylprednisolone after 3h of confirmation of BD and MP, rats treated with methylprednisolone after 3h of BD confirmation.

pg/mL	Sham	BD	MP/E2	MP	*P (Kruskal-Wallis)*
**IL-1β**	387.1 ± 127.4	505.2 ± 95.32	327.7 ± 97.75	273.9 ± 21.17	*0.4939*
**IL-6**	281.3 ± 88.28	937.1 ± 302.3	44.47 ± 17.64*	55.68 ± 22.45*	*0.0003*
**VEGF**	36.59 ± 9.274*	7.948 ± 2.997	1.571 ± 0.071*	5.742 ± 1.706	*0.0006*
**TNF-α**	48.67 ± 17.88	40.45 ± 14.87	39.57 ± 9.954	33.90 ± 8.698	*0.9301*
**CINC-1**	41.40 ± 9.127	23.96 ± 3.838	27.40 ± 5.260	24.86 ± 4.913	*0.3761*
**NO_x_^-^**	98.55 ± 24.93	186.2 ± 62.50	125.5 ± 17.28	93.27 ± 5.949	*0.1582*

Data expressed as mean ± SEM from 6-8 animals per group. *p<0.05 in relation to the BD group. IL. interleukin; VEGF. vascular endothelial growth factor; TNF- α. tumor necrosis factor alpha; CINC. cytokine-induced neutrophil chemoattractant; NO. nitric oxide.

### 3.4 Pulmonary inflammation

To evaluate pulmonary inflammation IL-1β, IL-6, TNF-α, and VEGF were quantified in lung tissue and explant.

#### 3.4.1 IL-1β

**Figure 4:**
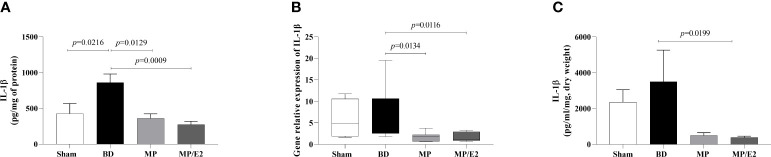
Quantification of IL-1β in lung homogenate **(A)** and explant **(C)** and gene expression in lung tissue **(B)**. Sham, false-operated rats; BD, rats submitted to brain death; MP, rats treated with methylprednisolone (MP) after 3h of confirmation of BD and MP/E2, rats treated with 17β-estradiol (E2) and methylprednisolone after 3h of confirmation of BD. Data expressed as mean ± SEM from 8 animals **(A, C)**. Data expressed as median and 95th percentile from 4-8 animals **(B)**. **(A)** p^(Kruskal Wallis)^=0.0057, **(B)** p^(Kruskal Wallis)^=0.0202, **(C)** p^(Kruskal Wallis)^=0.0295.

After BD, IL-1β was increased in lung homogenate and both MP and MP/E2 groups presented lower values. Also, both treatments were effective in reducing gene expression. Whereas in the explant, IL-1β was only reduced in the MP/E2 group (**Figure 4**).

#### 3.4.2 IL-6

**Figure 5:**
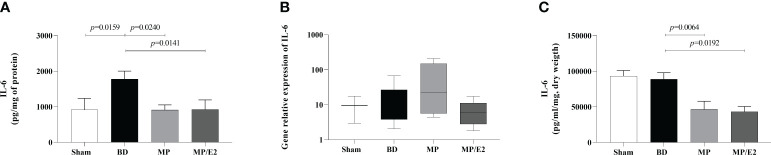
Quantification of IL-6 in lung homogenate **(A)** and explant **(C)** and gene expression in lung tissue **(B)**. Sham, false-operated rats; BD, rats submitted to brain death; MP, rats treated with methylprednisolone (MP) after 3h of confirmation of BD and MP/E2, rats treated with 17β-estradiol (E2) and methylprednisolone after 3h of confirmation of BD. Data expressed as mean ± SEM from 8 animals **(A, C)**. Data expressed as median and 95th percentile from 4-8 animals **(B)**. **(A)** p^(Kruskal Wallis)^=0.0288, **(B)** p^(Kruskal Wallis)^=0.2864, **(C)** p^(Kruskal Wallis)^=0.0027.

IL-6 was significantly increased in lung homogenate after BD and both the associated and the isolated treatments reduced protein expression in homogenate and explant. However, treatments had no differences in gene expression. There was no difference between Sham and BD groups in gene expression and explant (**Figure 5**).

#### 3.4.3 TNF-α

**Figure 6:**
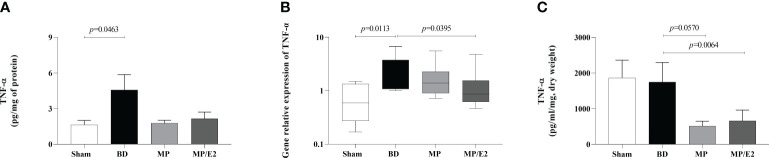
Quantification of TNF-α in lung homogenate **(A)** and explant **(C)** and gene expression in lung tissue **(B)**. Sham, false-operated rats; BD, rats submitted to brain death; MP, rats treated with methylprednisolone (MP) after 3h of confirmation of BD and MP/E2, rats treated with 17β-estradiol (E2) and methylprednisolone after 3h of confirmation of BD. Data expressed as mean ± SEM from 8 animals **(A, C)**. Data expressed as median and 95th percentile from 5-8 animals **(B)**. **(A)** p^(Kruskal Wallis)^=0.1992, **(B)** p^(Kruskal Wallis)^=0.0466, **(C)** p^(Kruskal Wallis)^=0.0369.

BD increased both gene and protein expression in lung tissue, with no change in the explant. Moreover, MP/E2 treatment reduced gene expression of TNF-α and both MP and MP/E2 significantly reduced this cytokine in explant (**Figure 6**).

#### 3.4.4 VEGF

**Figure 7:**
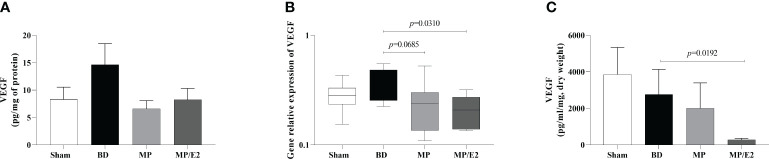
Quantification of VEGF in lung homogenate **(A)** and explant **(C)** and gene expression in lung tissue **(B)**. Sham, false-operated rats; BD, rats submitted to brain death; MP, rats treated with methylprednisolone (MP) after 3h of confirmation of BD and MP/E2, rats treated with 17β-estradiol (E2) and methylprednisolone after 3h of confirmation of BD. Data expressed as mean ± SEM from 8 animals **(A, C)**. Data expressed as median and 95th percentile from 6-8 animals **(B)**. **(A)** p^(Kruskal Wallis)^=0.4636, **(B)** p^(Kruskal Wallis)^=0.1275, **(C)** p^(Kruskal Wallis)^=0.0616.

In regards to VEGF, the MP/E2 group presented a reduction in gene expression. Also, in explant analyses, overall lower values were observed in the MP/E2 group in comparison to others (**Figure 7**).

### 3.5 Leukocyte infiltrates

To evaluate leukocyte migration from the microcirculation to the lung parenchyma and airways, we quantified total and differential cell counts in BAL. Additionally, in the lung parenchyma, MPO activity and protein expression were evaluated. Quantification of CINC-1 levels in lung homogenate and explant was also performed. In parallel, gene and protein expression of ICAM were analyzed.

#### 3.5.1 Bronchoalveolar lavage infiltrate

**Figure 8:**
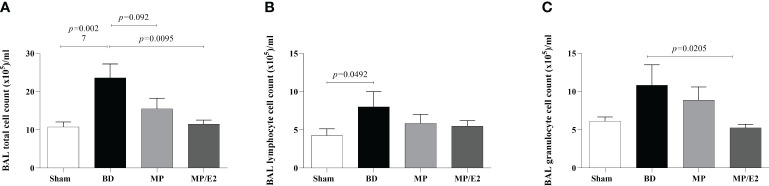
Total **(A)** and differential **(B, C)** number of cells present in bronchoalveolar lavage. Sham, false-operated rats; BD, rats submitted to brain death; MP, rats treated with methylprednisolone (MP) after 3h of confirmation of BD and MP/E2, rats treated with 17β-estradiol (E2) and methylprednisolone after 3h of confirmation of BD. Data expressed as mean ± SEM from 8 animals. **(A)** p^(Kruskal Wallis)^=0.0139, **(B)** p^(Kruskal Wallis)^=0.2327, **(C)** p^(Kruskal Wallis)^=0.1015.

There was an increase of total infiltrated leukocytes to the alveoli in the BD group and a reduction in the MP/E2 group. Concerning the differential analyses, lymphocytes were increased after BD compared with Sham, with no change in the treatment. Moreover, regarding granulocytes, there was a reduction only in the MP/E2 group (**Figure 8**).

#### 3.5.2 Myeloperoxidase

**Figure 9:**
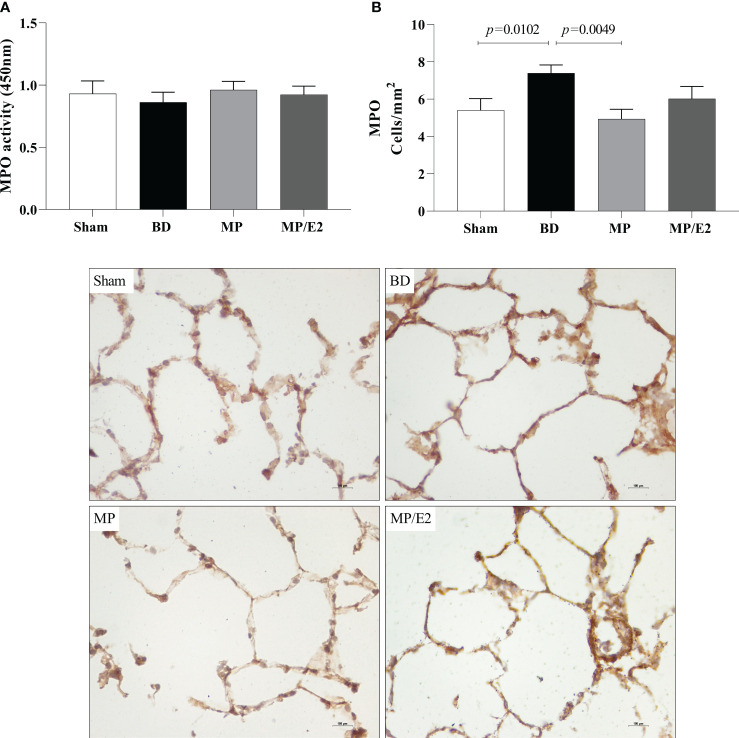
Enzymatic activity **(A)** and protein expression (immunohistochemistry) **(B)** of myeloperoxidase (MPO) in lung tissue. Sham, false-operated rats; BD, rats submitted to brain death; MP, rats treated with methylprednisolone (MP) after 3h of confirmation of BD and MP/E2, rats treated with 17β-estradiol (E2) and methylprednisolone after 3h of confirmation of BD. Data expressed as mean ± SEM from 5-8 animals. 1 section per animal and 10 areas per section were analyzed. The photomicrographs (x20) are representative of protein expression on each group. **(A)** p^(Kruskal Wallis)^=0.3400; **(B)** p^(Kruskal Wallis)^=0.0191.

Regarding protein expression of MPO, there was an increase of stained cells in the BD group in comparison to Sham and a decrease in the MP group. There were no significant differences among the groups concerning enzymatic activity (**Figure 9**).

#### 3.5.3 CINC-1, ICAM-1 and VCAM-1

**Figure 10:**
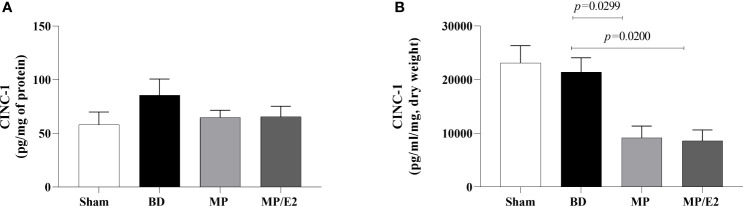
Quantification of CINC-1 in lung homogenate **(A)** and explant **(B)**. Sham, false-operated rats; BD, rats submitted to brain death; MP, rats treated with methylprednisolone (MP) after 3h of confirmation of BD and MP/E2, rats treated with 17β-estradiol (E2) and methylprednisolone after 3h of confirmation of BD. Data expressed as mean ± SEM from 5-8 animals per group **(A, B)**. **(A)** p^(Kruskal-Wallis)^=0.6090, **(B)** p^(Kruskal Wallis)^=0.0075.

**Figure 11:**
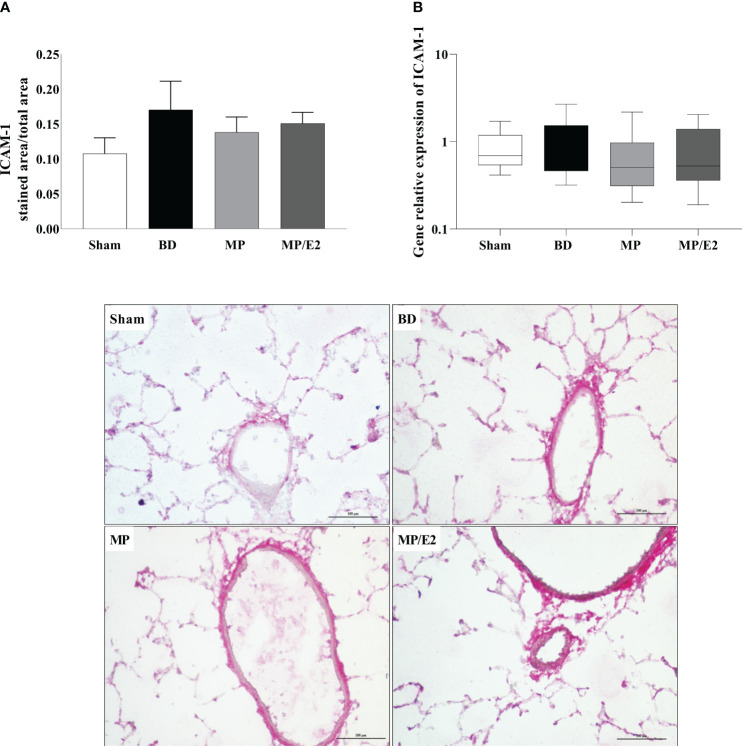
Protein **(A)** (immunohistochemistry) and gene **(B)** expression of ICAM-1. Sham, false-operated rats; BD, rats submitted to brain death; MP, rats treated with methylprednisolone (MP) after 3h of confirmation of BD and MP/E2, rats treated with 17β-estradiol (E2) and methylprednisolone after 3h of confirmation of BD. Data expressed as mean ± SEM from 5-8 animals per group **(A)**. Data expressed as median and 95th percentile from 6-8 animals **(B)**. 1 section per animal and 10 areas per section were analyzed. The photomicrographs (x20) are representative of protein expression on each group. **(A)** p^(Kruskal Wallis)^=0.6009; **(B)** p^(Kruskal Wallis)^=0.7960.

**Figure 12:**
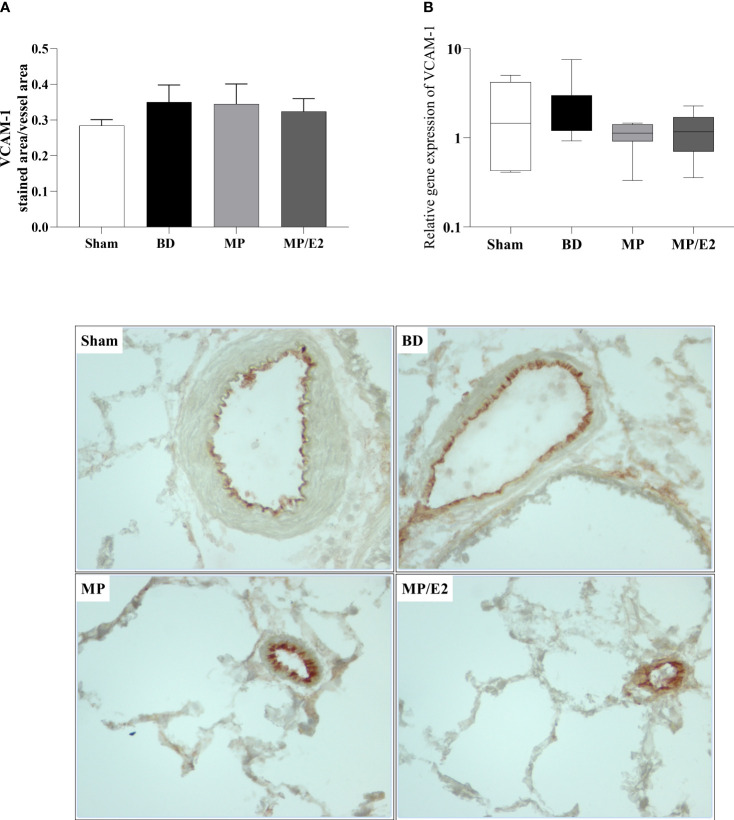
Protein **(A)** (immunohistochemistry) and gene **(B)** expression of VCAM-1. Sham, false-operated rats; BD, rats submitted to brain death; MP, rats treated with methylprednisolone (MP) after 3h of confirmation of BD and MP/E2, rats treated with 17β-estradiol (E2) and methylprednisolone after 3h of confirmation of BD. Data expressed as mean ± SEM from 5-8 animals per group **(A)**. Data expressed as median and 95th percentile from 6-8 animals **(B)**. 1 section per animal and 10 areas per section were analyzed. The photomicrographs (x20) are representative of protein expression on each group. **(A)** p^(Kruskal Wallis)^=0.7298; **(B)** p^(Kruskal Wallis)^=0.3855.

To further analyze leukocyte chemotaxis, early and late release of CINC-1 were quantified in lung homogenate and explant (24h after BD), no difference was observed in lung homogenate, however, both treatments were able to reduce CINC-1 in explant samples (**Figure 10**). Gene and protein expression of the adhesion molecules ICAM-1 (**Figure 11**) and VCAM-1 (**Figure 12**) were also evaluated in lung tissue, but no significant differences were found in both ICAM-1 and VCAM-1 analyses.

### 3.6 Analyzes of inducible and endothelial nitric oxide synthase protein and gene expression

**Figure 13:**
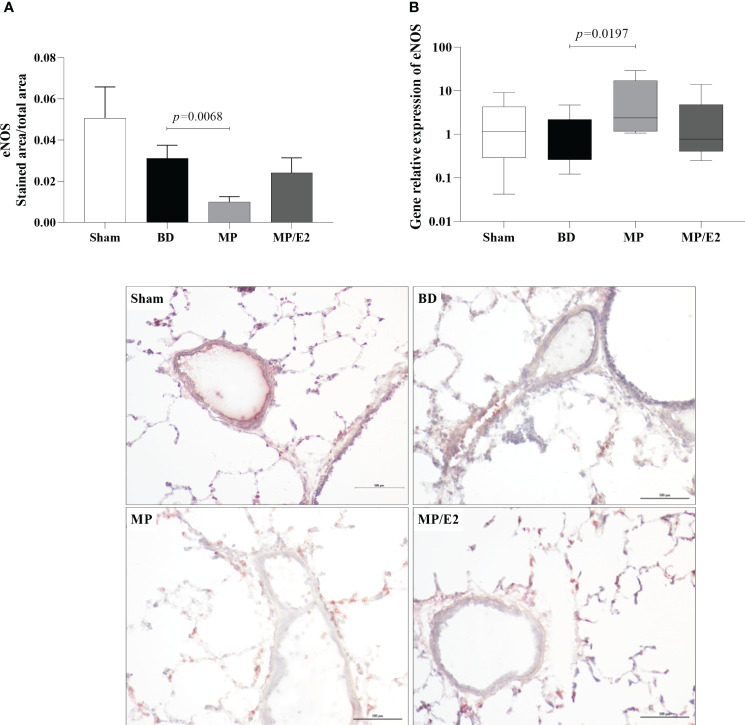
Protein **(A)** (immunohistochemistry) and gene expression of eNOS **(B)**. Sham, false-operated rats; BD, rats submitted to brain death; MP, rats treated with methylprednisolone (MP) after 3h of confirmation of BD and MP/E2, rats treated with 17β-estradiol (E2) and methylprednisolone after 3h of confirmation of BD. Data expressed as mean ± SEM from 5 animals per group **(A)**. Data expressed as median and 95th percentile from 6-8 animals **(B)**. 1 section per animal and 10 areas per section were analyzed. The photomicrographs (x20) are representative of protein expression on each group. **(A)** p^(Kruskal-Wallis)^=0.0040, **(B)** p^(Kruskal Wallis)^=0.0962.

**Figure 14:**
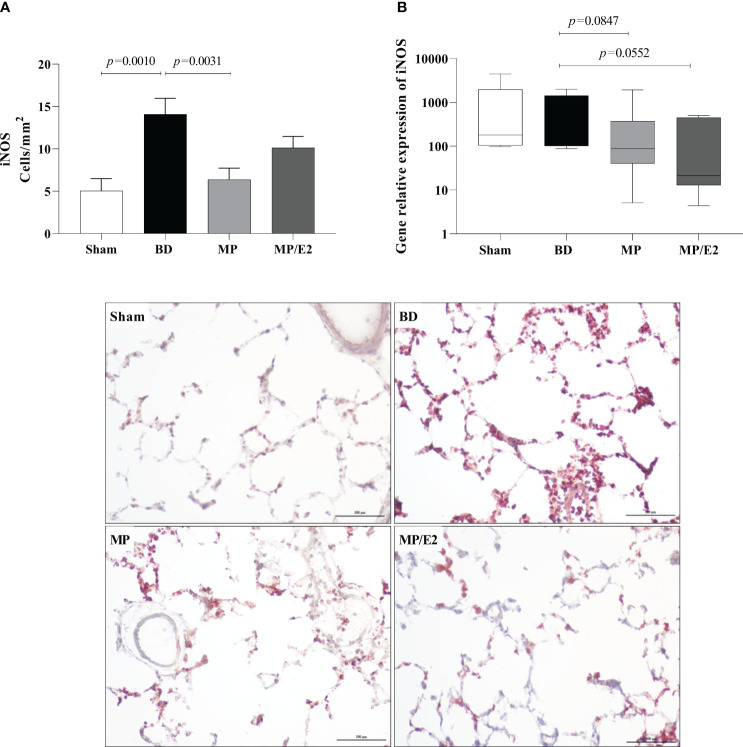
Protein **(A)** (immunohistochemistry) and gene expression of iNOS **(B)**. Sham, false-operated rats; BD, rats submitted to brain death; MP, rats treated with methylprednisolone (MP) after 3h of confirmation of BD and MP/E2, rats treated with 17β-estradiol (E2) and methylprednisolone after 3h of confirmation of BD. Data expressed as mean ± SEM from 5 animals per group **(A)**. Data expressed as median and 95th percentile from 6-8 animals **(B)**. 1 section per animal and 10 areas per section were analyzed. The photomicrographs (x20) are representative of protein expression on each group. **(A)** p^(Kruskal Wallis)^=0.0019, **(B)** p^(Kruskal Wallis)^=0.0950.

Regarding protein, there is a decrease in the expression of eNOS in the MP group compared to BD. In contrast, there is an increase of eNOS in the MP group in gene expression (**Figure 13**). In iNOS analyses, there was an increase in the BD group and a reduction only in the MP-treated group in protein expression. Regarding gene expression, although there is no difference between the Sham and BD groups, there is a reduction after both treatments (**Figure 14**).

### 3.7 Quantification of NOx^–^

**Figure 15:**
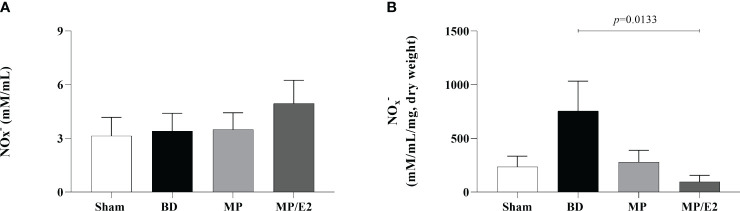
Quantification of nitric oxide metabolites in homogenate **(A)** and explant **(B)**. Sham, false-operated rats; BD, rats submitted to brain death; MP, rats treated with methylprednisolone (MP) after 3h of confirmation of BD and MP/E2, rats treated with 17β-estradiol (E2) and methylprednisolone after 3h of confirmation of BD. Data expressed as mean ± SEM from 6-8 animals per group. **(A)** p^(Kruskal-Wallis)^ =0.9138, **(B)** p ^(Kruskal Wallis)^ =0.0845.

To indirectly determine nitric oxide’s presence, nitrites and nitrate were quantified by quantification of NOx^–^ in lung homogenate and explant samples. In the explant, there was a reduction of NOx^–^ in the MP/E2 group in comparison to BD and no changes were observed in the tissue homogenates (**Figure 15**).

## 4 Discussion

This study investigated if E2 and MP association had positive effects in modulating inflammation in females after BD, and we observed that the hormone combination was able to positively regulate inflammation, especially on leukocyte infiltration and endothelial health. Relevant aspects of systemic and pulmonary evaluation were analyzed, such as systemic and local quantification of inflammatory mediators, leukocyte infiltration to the lung parenchyma and airways, as well as adhesion molecule expression. With our data, we observed that the treatments were able to significantly increase corticosterone and estradiol, to levels higher than non-BD controls.

Our model is based on the fast induction of BD and after traumatic brain injury, increased intracranial pressure leads to herniation of the brain stem. We observed a rapid increase in mean arterial blood pressure just after balloon insufflation, followed by a period of hypotension. Several guidelines propose the use of catecholamines in the hemodynamic management of the donor. However, studies suggest that the use of norepinephrine can be detrimental to the organs, causing increased pulmonary permeability. The use of corticoid supplementation has shown positive effects in decreasing the donor’s need for catecholamines (23). Our results, however, show that administration of methylprednisolone and E2 combined was able to reinstate MAP levels close to Sham animals one hour after the start of treatment, while methylprednisolone alone did not present the same effect. Additionally, recent work in a sepsis model showed that treatment of female animals with E2 increased the expression of corticoid receptor α (GRα) in vascular smooth cells. This receptor was associated with glucocorticoid activation of vascular activity and the upregulation of this receptor by E2, enhanced corticoid-positive action in vascular dysfunction (24).

Another known physiological imbalance triggered by BD is inflammation. High IL-6 levels were correlated with early allograft dysfunction after transplantation, while lower levels were associated with improved graft survival (25). In our model, BD indeed increased IL-6 levels in the serum, which was reduced by both treatments; the same reduction was observed in the long-term analysis of IL-6 explant levels, indicating that after transplant, IL-6 levels could be controlled by both treatments. Corticoids have notorious anti-inflammatory properties by inhibiting several pro-inflammatory cytokines, including IL-6 (26). Moreover, high concentrations of E2 also suppress IL-6 expression by down-regulating NK-κB (27).

Lungs are most vulnerable to the detrimental effects of BD, reflecting the low transplant rate for this organ. The sympathetic storm leads to the disruption of endothelial cells and the alveolar barrier (23), and, acute systemic inflammation leads to the infiltration of activated neutrophils to the lungs, leading to tissue injury (28, 29). Previous studies from our group have shown that BD increases inflammatory markers in lung tissue in the short and long-term and that E2 presents anti-inflammatory properties that could attenuate lung injury (13, 14). The same behavior can be observed in this study after 6h of BD by the increased levels of inflammatory markers in lung homogenate and lung culture samples 24 hours after the experiment. Both treatments were effective in reducing protein and gene expression at the moment of organ procurement and 24 hours later. These results indicate that the treatment of the donor, with MP alone or with MP combined with E2, could have a positive effect on the graft in the short and long term.

Moreover, to further evaluate the inflammatory response in the lungs, we investigated the leukocyte infiltration to the parenchyma and airway. Donor leukocyte in the lung is directly involved in acute rejection in the recipient. The migration of donor infiltrate cells to recipient lymph nodes leads to the activation of naïve T cells, resulting in allograph rejection (30). MPO results show that there was an increase of infiltrate neutrophils in the lung parenchyma and that the treatment with MP showed lower number of neutrophils. However, no differences were observed among the groups regarding the activity of those cells. Even though no changes were found in adhesion molecules, a higher number of leukocytes were present in bronchoalveolar lavage samples, primarily of granulocytes, and the associated treatment was able to reduce their number in the airways. In the lungs, cytokines and chemokines are released from damaged epithelial cells, as well as resident macrophages after injury. Leukocyte migration to the lung parenchyma follows a specific chain of events that are believed to be independent of adhesion molecules, such as ICAM-1 and VCAM-1. High ICAM-1 levels are expressed in lung vasculature in a steady state and cell migration may be dependent more of chemokine gradients. Indeed, in a model of LPS challenge in mice, blockage of ICAM-1 did not affect neutrophil recruitment to the lung parenchyma or bronchoalveolar space (28, 31). Activated neutrophils present a slower transit time in the lung vasculature, which is believed to stimulate neutrophil migration through the endothelial cell junctions. Once in the lung parenchyma, neutrophils are attracted to the airways and secrete proteases, like metalloproteinase-9 (MMP-9), to migrate through the lung interstitium (32, 33). Here, we show that treatment with MP alone did not prevent the migration of cells from the lung parenchyma to the bronchoalveolar space. Previous studies have shown that estradiol treatment was able to reduce cell migration to the airways, by reducing chemokines such as MIP-1, MIP-2 and CINC-1, along with reduction in MMP-9 activity (13), and depletion of donor cells in lungs have been shown to improve transplant results (34). Previous and current results suggest an estradiol-dependent mechanism in modulating neutrophil activation in the lung parenchyma of females. These point to the use of estradiol in the management of female BD donors as a therapeutic option to improve transplant outcomes, by reducing leukocyte trafficking to the lung and thus modulating the recipient immunogenic response and allograft rejection.

Additionally, to evaluate the effect of both treatments in the endothelium, we analyzed protein and genomic expression of eNOS and iNOS, NO levels in lung homogenate and lung culture, as well as systemic and local VEGF concentrations. NO is a soluble gas with strong vasodilatory properties that acts in maintaining the homeostasis of the vascular bed. eNOS and iNOS are the main sintases responsible for NO production in the vasculature. iNOS expression is mediated by cytokines, mainly IL-1β, TNF-α, and IFN-γ, and a high concentration of iNOS-derived NO is involved in the immune response and inflammation. eNOS is constitutively expressed in endothelial cells and is related to the maintenance of vascular tone by releasing nanomolar amounts of NO (35, 36). Previous studies have shown that females after BD present higher expression of eNOS compared to males (37), which was associated with high estradiol levels before BD induction (11). E2 is known to upregulate eNOS expression by both genomic and non-genomic pathways. E2 binding to ERβ was associated with increased expression of eNOS mRNA, while activation of ERα led to an acute increase in eNOS activity (38, 39). Also, estradiol treatment after BD in both males and females has been shown to modulate eNOS expression (11, 13, 14) and was associated with increased flow in the mesenteric microcirculation (40). Our results corroborate those findings by showing that the MP/E2 group was able to prevent further eNOS decrease, while MP alone presents significantly lower values in comparison to BD. Moreover, gene expression of iNOS presented lower values in both treated groups, however, NO quantification in explant samples after 24h suggested that the associated treatment has a long-term effect in reducing NO release.

Likewise, in VEGF analyses, we observed that, overall, all groups that underwent BD induction presented lower levels of VEGF in comparison to Sham. Higher expression of VEGF in Sham animals could be related to the maintenance of the anesthetic state with isoflurane during the 6 hours of experiment, as isoflurane exposure has been shown to increase VEGF mRNA expression, even in levels as low as 2% (41). Regarding the treatment groups, MP/E2 treatment was able to reduce both systemic and local expression of VEGF. VEGF actions are related to enhanced permeability, increased leukocyte migration, and activation of angiogenic processes (42). Corticoids are widely used in different diseases to reduce VEGF levels (43, 44), however, E2 is a known inducer of VEGF mRNA expression (45). Thus, our results show that MP and E2 association has a positive effect compared to MP alone, suggesting a synergic effect of both hormones in modulating vascular permeability.

This investigation has certain limitations. The time point after 6 hours could limit the analyses of later outcomes. However, explant results provide us with an overview of the lung inflammatory profile 24 hours after procurement. Moreover, we chose to administer a continuous infusion of both hormones after 3 hours of BD. The increased concentration of both hormones for this period could potentially reduce gene and protein expression of ER and GPER (E2 rapid response receptor) and GR, interfering with the receptor response.

In conclusion, this study brings new insights into the role of sex hormones in the management of the BD donor. Showing that E2 association with already well-known anti-inflammatory drugs, like methylprednisolone, could have potentially positive effects on the inflammatory process triggered by BD in females, by modulating the hemodynamics balance, as well as leukocyte infiltration and maintenance of endothelial and vascular homeostasis.

## Data availability statement

The raw data supporting the conclusions of this article will be made available by the authors, without undue reservation.

## Ethics statement

The animal study was approved by Faculdade de Medicina da Universidade de São Paulo Ethic Committee for Research Projects. The study was conducted in accordance with the local legislation and institutional requirements.

## Author contributions

MV: Formal analysis, Funding acquisition, Investigation, Methodology, Writing – original draft. LA: Methodology, Visualization, Writing – review & editing. RA: Methodology, Visualization, Writing – review & editing. FR: Investigation, Validation, Writing – review & editing. IR: Writing – review & editing, Methodology, Visualization. CC: Conceptualization, Supervision, Visualization, Writing – review & editing. HL: Conceptualization, Investigation, Supervision, Validation, Visualization, Writing – review & editing. LM: Conceptualization, Data curation, Formal analysis, Resources, Validation, Visualization, Writing – review & editing. AB: Conceptualization, Formal analysis, Funding acquisition, Investigation, Methodology, Project administration, Resources, Supervision, Validation, Writing – original draft.
